# In-depth transcriptomic analyses of LncRNA and mRNA expression in the hippocampus of APP/PS1 mice by Danggui-Shaoyao-San

**DOI:** 10.18632/aging.104068

**Published:** 2020-11-18

**Authors:** Zhenyan Song, Fuzhou Li, Chunxiang He, Jingping Yu, Ping Li, Ze Li, Miao Yang, Shaowu Cheng

**Affiliations:** 1 Key Laboratory of Hunan Province for Integrated Traditional Chinese and Western Medicine on Prevention and Treatment of Cardio-Cerebral Diseases, Hunan University of Chinese Medicine, Changsha 410208, Hunan, China

**Keywords:** Alzheimer's disease, Dangui-Shaoyao-San, LncRNA, APP/PS1 mice, transcriptomic analyses

## Abstract

Alzheimer’s disease (AD) is an age-related neurodegenerative disease with a high incidence worldwide, and with no medications currently able to prevent the progression of AD. Danggui-Shaoyao-San (DSS) is widely used in traditional Chinese medicine (TCM) and has been proven to be effective for memory and cognitive dysfunction, yet its precise mechanism remains to be delineated. The present study was designed to investigate the genome-wide expression profile of long non-coding RNAs (LncRNAs) and messenger RNAs (mRNAs) in the hippocampus of APP/PS1 mice after DSS treatment by RNA sequencing. A total of 285 differentially expressed LncRNAs and 137 differentially expressed mRNAs were identified (fold-change ≥2.0 and *P* < 0.05). Partial differentially expressed LncRNAs and mRNAs were selected to verify the RNA sequencing results by quantitative polymerase chain reaction (qPCR). A co-expression network was established to analyze co-expressed LncRNAs and genes. Gene ontology (GO) and Kyoto Encyclopedia of Genes and Genomes (KEGG) analyses were used to evaluate the biological functions related to the differentially co-expressed LncRNAs, and the results showed that the co-expressed LncRNAs were mainly involved in AD development from distinct origins, such as APP processing, neuron migration, and synaptic transmission. Our research describes the lncRNA and mRNA expression profiles and functional networks involved in the therapeutic effect of DSS in APP/PS1 mice model. The results suggest that the therapeutic effect of DSS on AD involves the expression of LncRNAs. Our findings provide a new perspective for research on the treatment of complex diseases using traditional Chinese medicine prescriptions.

## INTRODUCTION

Alzheimer’s disease (AD), the most common type of dementia in aging populations, has a complicated etiology, involves several nervous dysfunctions, and represents a challenge in health care systems [1]. Currently, the molecular mechanisms underlying AD pathogenesis remain unknown [2]. Although many drugs have been developed to treat AD, none slows or prevents the progression of AD [3]. AD is a complex disease caused by multiple pathways; hence, the treatment principle of monotherapy acting on a single target is not appropriate. Conversely, the holistic view of traditional Chinese medicine (TCM) regarding the treatment of multiple components and multiple targets provides a bright prospect for the prevention and treatment of AD [4]. A TCM formulation (prescription or fangji in Chinese) Danggui-Shaoyao-San (DSS), also called Dangguijakyak-san or Toki-shakuyaku-san in Japanese, has been used for neurodegenerative diseases in China for more than 2,000 years. Clinically, a large amount of evidence supports the therapeutic effect of DSS on AD through limiting neuronal damage, enhancing cognitive behavior, inhibiting the deposition of amyloid-β (Aβ) in the hippocampus, and reducing neuroinflammation [5–8]. However, further investigation of the molecular mechanism of the therapeutic effect of DSS on AD is required to propose effective treatment strategies.

Long non-coding RNAs (LncRNAs) are non-coding RNAs with a length of more than 200 nucleotides that are not translated into protein [9]. LncRNAs play an important role in many biological and pathological processes such as the dosage compensation effect, epigenetic regulation, cell cycle regulatory, and post-transcriptional regulation [10]. Recently, the role of LncRNAs in AD has attracted wide attention. It was reported that β-secretase 1 (BACE1)-antisense transcript (BACE1-AS) is an LncRNA that is highly expressed in AD [11]. LncRNA 51A increases β-amyloid formation by selectively splicing SORL1 variant A and is upregulated in AD [12]. Moreover, studies have predicted that LncRNA may be embedded in the genes *APP*, *APOE,* and *PSEN1* affecting the pathological process of AD [13]. However, knowledge of the role of LncRNAs in AD is limited.

In the present study, we investigated the differences in LncRNA and mRNA expression in the hippocampus of APP/PS1 double transgenic mice treated with DSS or untreated, using RNA sequencing, and LncRNA and mRNA co-expression networks were constructed. Bioinformatics methods including gene ontology (GO) and Kyoto Encyclopedia of Genes and Genomes (KEGG) enrichment analyses were used to identify differentially expressed genes. The RNA sequencing results were further verified using quantitative polymerase chain reaction (qPCR) to detect LncRNA and mRNA expression in these mice.

## RESULTS

### Quality control of DSS freeze-drying powder

**Figure 1:**
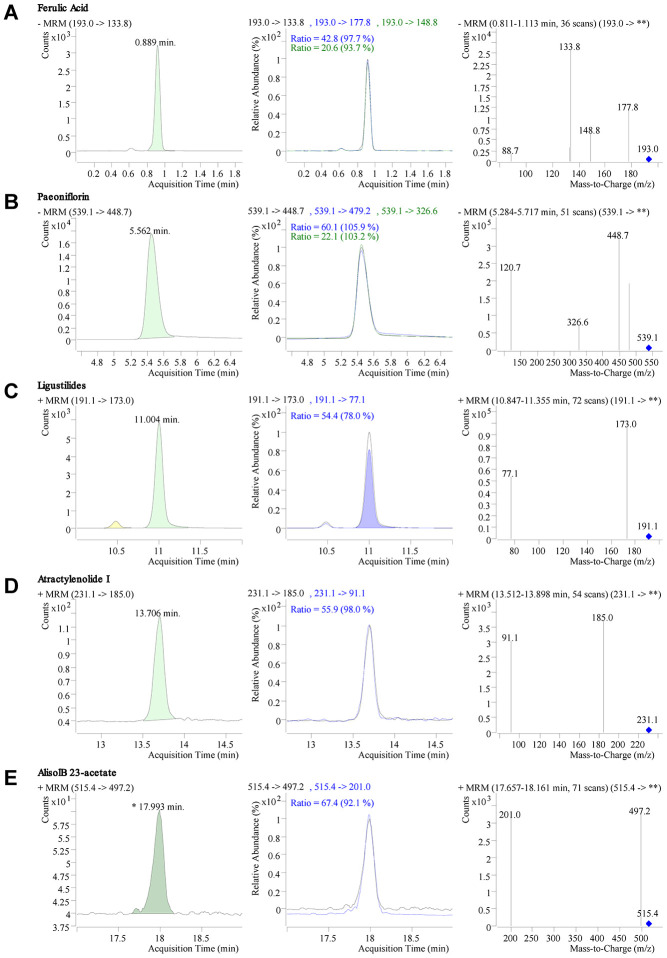
**LC-MS/MS chromatogram and mass spectrometry of Danggui-Shaoyao-San (DSS).** (**A**–**E**) Retention time, chromatogram, and mass spectrogram of ferulic acid (**A**), paeoniflorin (**B**), ligustilide (**C**), atractylenolide I, (**D**) and alisol B 23-acetate (**E**) in DSS.

Five quality standards (ferulic acid, paeoniflorin, ligustilide, atractylenolide I, and alisol B 23-acetate) in DSS freeze-dried powder were identified by liquid chromatography-tandem mass spectrometry (LC-MS/MS). The chromatogram results showed that the retention time of ferulic acid, paeoniflorin, ligustilide, atractylenolide I, and alisol B 23-acetate were 0.889min, 5.562min, 11.004 min, 13.706 min, and 17.993 min, respectively (Figure 1A–1E). The quantitative results of the mass spectrometry showed that the content of ferulic acid, paeoniflorin, ligustilide, atractylenolide I, and alisol B 23-acetate in DSS were 148.2 mg/kg, 5320 mg/kg, 400.7 mg/kg, 7.2 mg/kg, and 26.7 mg/kg, respectively(Figure 1A–1E).

### Effect of DSS on learning and memory deficits in APP/PS1 mice

**Figure 2:**
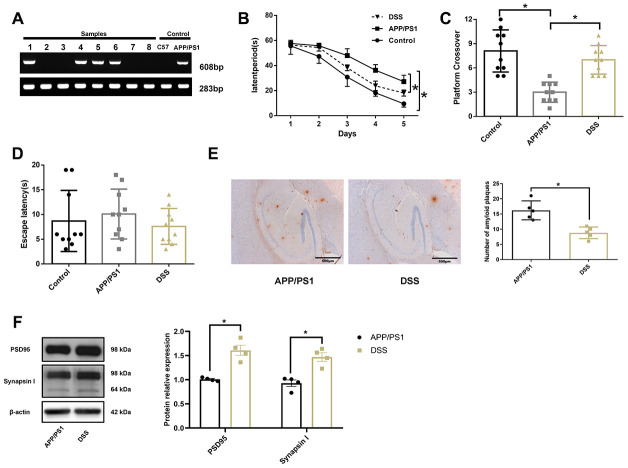
**Danggui-Shaoyao-San rescued learning and memory deficits in APP/PS1 transgenic mice.** (**A**) The genotype identification of APP/PS1 double transgenic mice; (**B**) Escape latency during the acquisition phase of the Morris water maze test; (**C**) The number of crossings over the previously hidden platform area in the Morris water maze test; (**D**) Escape latency during the visible platform phase of the Morris water maze test. N = 10 mice/group; Age = 7 months; Data are represented as mean ± standard deviation (SD), *, *P*< 0.05. (**E**) Immunohistochemistry of amyloid-β in the brain. N = 5 mice/group; Data are represented as mean ± SD, *, *P*< 0.05. (**F**) The protein expression of synaptic markers. N = 4 mice/group; Data are represented as mean ± SD, *, *P*< 0.05.

The offspring mice were weaned at the age of 3 weeks, and tail biopsies were collected for genotype identification on the second day after weaning. We used the gene-specific primer PS1 (608 bp) and reference gene primer GAPDH (283 bp) to detect mouse genotype, as shown in Figure 2A. The samples numbered 1, 4, 5, 6, and the positive control sample had a bright band at around 283 bp and 608 bp, respectively. Other specimens and the negative control sample had only one bright band at around 283 bp. APP/PS1 mice contain human transgenes for both *APP* bearing the Swedish mutation and *PSEN1* containing an L166P mutation, both under the control of the Thy1 promoter [14]. Therefore, the samples of mice amplified with *PS1* genes were thought to have been successfully transfected with *APP* and *PS1*. A Morris water maze test was conducted on mice at 7 months of age, as shown in Figure 2B–2D. Wild-type C57BL/6J mice (control) easily learned the hidden location during a 5-day trial, but APP/PS1 mice showed an inability to find the platform. Notably, the DSS-treated APP/PS1 mice showed significant improvement in learning and memory function compared with the untreated APP/PS1 mice, as evidenced by a reduction in the latency of avoidance (Figure 2B). Similar to the control mice, DSS-treated APP/PS1 mice crossed over the previous platform location more frequently than untreated APP/PS1 mice (Figure 2C). In the visible platform version of the Morris water maze test, DSS-treated and untreated APP/PS1 mice demonstrated similar behaviors (Figure 2D).

To further confirm the improvement of cognitive function in the APP/PS1 transgenic mice by DSS, immunohistochemistry was used to detect Aβ deposition in the hippocampal region of the mouse (Figure 2E). Consistent with other tests, immunohistochemical analysis showed that Aβ deposition in the hippocampus of DSS-treated APP/PS1 mice was significantly lower than that of APP/PS1 mice (*P* < 0.05). The synapse is the main part of neurons to transmit information, and the synaptic markers mainly include postsynaptic density protein 95 (PSD95) and synaptophysin. PSD95 is the most important and abundant scaffold protein on the postsynaptic membrane, which mainly exists in the mature excitatory glutamate synapses. PSD95 is necessary for the activity and stability of receptors on the postsynaptic membrane, which is involved in the regulation of the number of synapses during development and the promotion of the formation of synapses [15, 16]. synaptophysin is a calcium-binding protein that is specific to the synaptic vesicle membrane of presynaptic components and can indirectly reflect the number, distribution, and density of synapses [17, 18]. Western blotting results showed (Figure 2F) that the expression of PSD95 and synapsin Ι in hippocampus of APP/PS1 mice treated by DSS were significantly higher than those of APP/PS1 mice (*P* < 0.05). These results suggest that 7-month-old APP/PS1 transgenic mice develop AD spontaneously and that DSS improved the learning and memory deficits in APP/PS1 mice.

### Differential expression analysis of mRNAs and LncRNAs

**Figure 3:**
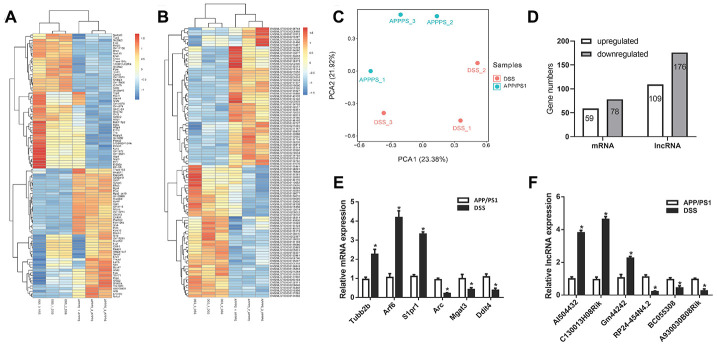
**Differential expression analysis of messenger RNA (mRNAs) and long non-coding RNAs (LncRNAs) in Danggui-Shaoyao-San (DSS)-treated and untreated APP/PS1 double transgenic mice.** (**A**) Heatmap analysis of differentially expressed mRNAs, DSS-treated VS. APP/PS1 mice; (**B**) Heatmap analysis of differentially expressed LncRNA, DSS-treated VS. APP/PS1 mice; (**C**) Principal component analysis (PCA), DSS-treated VS. APP/PS1 mice; (**D**) Differentially expressed LncRNAs and mRNAs; (**E**) qPCR for mRNAs; (**F**) qPCR for LncRNA. N = 5 mice/group; Data are represented as mean ± standard error of mean (SEM), *, *P* <0.05 vs. APP/PS1 mice group.

RNA sequencing was used to detect the LncRNA expression profile in two groups (DSS-treated APP/PS1 mice and APP/PS1 mice). The transcriptome analysis results showed that 285 differentially expressed LncRNAs were identified, of which 109 were upregulated and 176 were downregulated. 110 of the differentially expressed LncRNAs passed the False Discovery Rate (FDR) thresholds with the corrected *P* < 0.05, of which 47 were upregulated and 63 were downregulated. In addition, it was found that 137 mRNAs were differentially expressed, including 59 upregulated and 78 downregulated (Figure 3D). These differentially expressed LncRNAs and mRNAs were shown as a heatmap (Figure 3A, 3B). Principal component analysis (PCA) showed that there were significant differences in gene expression profile between after DSS - treated and untreated in APP/PS1 mice (Figure 3C). However, intra-group differences still exist, and we should focus on those genes that are relatively high in expression. So, in order to verify the transcriptome analysis results, we randomly selected four LncRNAs and four mRNAs from differentially expressed LncRNA and mRNA transcripts for determining their expression using qPCR (Figure 3E, 3F). By comparison, we found that the qPCR results were similar to the RNA sequencing results.

### Co-expression network and functional analysis

**Figure 4:**
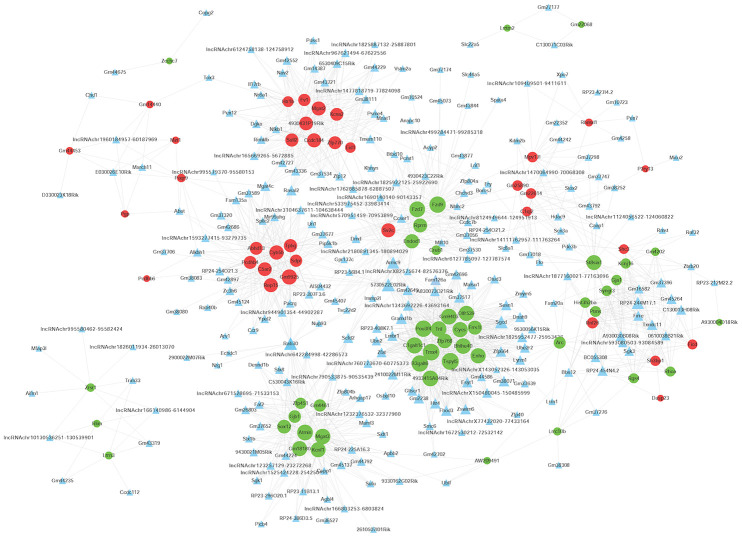
**Co-expression network of differentially expressed long non-coding RNAs (LncRNAs) and differentially expressed messenger RNAs (mRNAs).** The Pearson correlation coefficient between differentially expressed LncRNAs and mRNAs was calculated to construct the co-expression network, and the Pearson correlation coefficient ≥ 0.95 was selected. The circular nodes represent differentially expressed mRNAs (green: downregulated; red: upregulated). The triangular nodes represent differentially expressed LncRNAs. Connection line: co-expression between differentially expressed LncRNAs and mRNAs.

**Figure 5:**
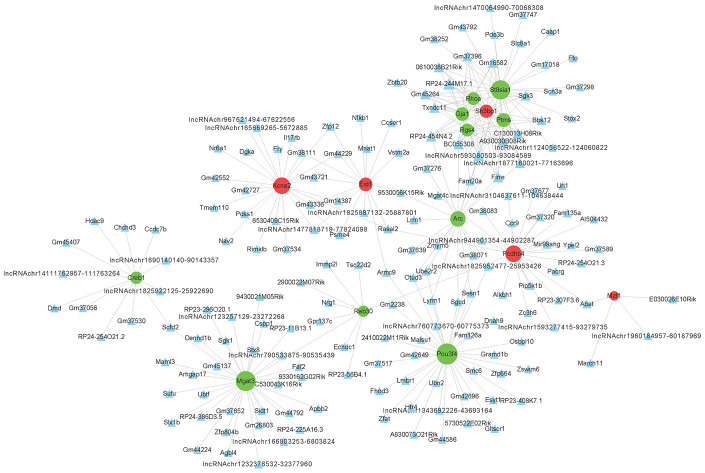
**Co-expression network of differentially expressed long non-coding (LncRNAs) and messenger RNAs (mRNAs) related to Alzheimer’s disease.** The construction method of the co-expression network is consistent with Figure 3.

To determine the function of differentially expressed LncRNAs, we investigated their correlation with each maladjusted mRNA. We identified 1528 co-expression relationships between 82 mRNAs and 242 LncRNAs (Figure 4). In addition, we also focused on the analysis of differentially expressed mRNAs related to AD (Table 1) and their co-expression relationship with LncRNA. The co-expression network is shown in Figure 5. Circular nodes represent mRNAs, the inverted triangle nodes represent LncRNAs, and the size of the node represent the degree of the node in the network. Usually, a high degree indicates an important node in the network; LncRNA:chr5:93080503-93084589, LncRNA A930030B08Rik, and LncRNA Firre showed the widest correlations (degree = 7), suggesting that they are involved in the regulation of multiple gene expressions. Similarly, mRNA Pou3f4 and mRNA Mgat3 were 34 and 30 degrees, respectively, indicating that their dysregulated expression profiles were associated with multiple LncRNAs.

**Table 1:** Differentially expressed mRNAs in hippocampus of APP/PS1 transgenic mice with Alzheimer disease.

**Gene name**	***P*-value**	**Log2(FC)**	**Description**
Gja1	0.00000	-3.88	gap junction protein, alpha 1
Arc	0.00001	-1.77	activity regulated cytoskeletal-associated protein
Creb1	0.00166	-1.46	cAMP responsive element binding protein 1
Eid1	0.00000	14.89	EP300 interacting inhibitor of differentiation 1
Mgat3	0.00000	-4.12	mannoside acetylglucosaminyltransferase 3
Rgs4	0.00000	-1.62	regulator of G-protein signaling 4
Rhoa	0.00012	-1.52	ras homolog family member A
St8sia1	0.02919	-1.34	ST8 alpha-N-acetyl-neuraminide alpha-2,8-sialyltransferase 1
Pou3f4	0.00210	-9.84	POU domain, class 3, transcription factor 4
Ptms	0.00000	-1.65	parathymosin

**Figure 6:**
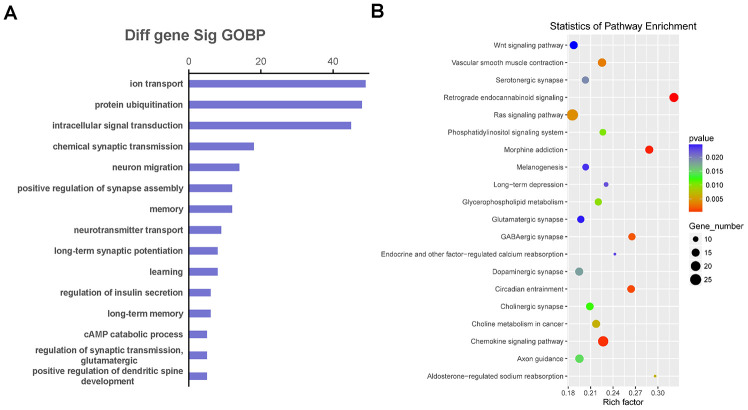
**The function and pathway analysis of co-expressed genes.** The gene ontology (GO) and Kyoto Encyclopedia of Genes and Genomes (KEGG) pathway enrichment analyses were performed on differentially expressed long non-coding RNA (LncRNA)-related genes to predict the potential biological processes and pathways affected by Danggui-Shaoyao-San-treated Alzheimer's disease mice. (**A**) The GO enrichment analysis (Biological Process) of differentially expressed genes co-expressed with differentially expressed LncRNAs; (**B**) The KEGG pathway enrichment analysis of differentially expressed genes co-expressed with differentially expressed LncRNAs.

We selected co-expressed genes for functional enrichment analysis to further explore the biological functions affected by the co-expression relationship of LncRNA and mRNA (Figure 6). The GO enrichment analysis results showed that the co-expressed genes were mainly involved in ion transport, protein ubiquitination, intracellular signal transduction, neuron migration, memory, learning, long-term synaptic potentiation, and regulation of glutamatergic synaptic transmission (Figure 6A). In addition, the pathway enrichment analysis results showed that the common enriched pathways related to co-expressed genes included the Wnt and Ras signaling pathways, glycerophospholipid metabolism, phosphatidylinositol signaling system, long-term depression, glutamatergic synapse, dopaminergic synapse, and cholinergic synapse (Figure 6B).

## DISCUSSION

For thousands of years, TCM has played an irreplaceable role in the Chinese medical system [19], and TCM has unique advantages in treating complex diseases such as AD [20, 21]. DSS is a TCM prescription with a significant effect on AD [22], but the mechanism of DSS for the treatment of AD is still unclear. In the present study, we studied the genome-wide expression profile of LncRNAs and mRNAs in DSS-treated and untreated APP/PS1 double transgenic mice using RNA sequencing. We identified 285 differentially expressed LncRNAs, including 109 upregulated and 176 downregulated between the DSS-treated and untreated APP/PS1 mice. Meanwhile, a total of 137 differentially expressed mRNAs were found, including 59 upregulated and 78 downregulated. We randomly selected some transcriptome analysis data for qPCR detection, and the expression differences of LncRNAs and mRNAs were consistent.

In order to investigate the therapeutic effect of DSS on AD, we screened genes related to AD from differentially expressed genes. These genes were mainly related to cell communication, neuronal development, synaptic transmission, synapse assembly, neuron migration, learning, or memory. These functions may be closely related to the DSS treatment of AD. For example, in neurobiology, activity-regulated cytoskeleton-associated protein (*ARC*) is an important marker of brain plasticity because of its activity regulation, localization, and utility [23]. *ARC* plays a negative regulatory role in gene expression, and increased *ARC* levels might impair memory-stabilizing processes [24]. The transcriptome analysis data showed that the ARC mRNA level in the DSS-treated mice was significantly lower than in untreated mice, suggesting that DSS improves functional disorders in *ARC* protein production. *SH3BP1*, another differential expressed gene, is a Rac1, Cdc42, and TC10-specific GTPase activating protein (GAP) protein, which specifically converts GTP-bound Rho-type GTPases including RAC1 and CDC42 in their inactive GDP-bound form. Recently, an mRNA transcript that is a partial fusion of *SH3BP1* and *CIN* gene products has been linked to AD, possibly through its effect on Rac1 inhibition and reactive oxygen species (ROS) generation [25]. Furthermore, RhoA-mediated activation of ROCK increases Aβ42 secretion via modulation of γ-secretase [26], and Rab family proteins involved in vesicle trafficking and thereby affects the trafficking and intracellular localization of APP [27]. MGAT3 is one of the most important enzymes involved in the regulation of the biosynthesis of glycoprotein oligosaccharides [28, 29]. In brain, addition of bisecting N-acetylglucosamine to BACE1 blocks its lysosomal targeting in response to oxidative stress and further degradation which increases its location to early endosome and the APP cleavage [28, 30]. Our results showed that DSS treatment could affect the small GTPase mediated signal transduction (*SH3BP1, RhoA* and *Rab* mRNA level) and reduce the MGAT3 mRNA level. Moreover, Aβ deposition in the hippocampus of DSS-treated APP/PS1 mice was significantly lower than that of APP/PS1 mice, suggesting that DSS decreases the level of Aβ partly by modulating APP processing.

A recent study found that only one-fifth of transcripts in the human genome is related to protein-coding genes, with non-coding RNA sequences accounting for the remaining four-fifths [31]. There has been considerable debate about whether LncRNAs were mislabeled and actually affected protein-encoding, but some LncRNAs have been found to encode for peptides with biologically significant function [32, 33]. The highest number of LncRNAs were found in non-coding RNAs, and the LncRNAs were expressed differently in different stages of development. Many association studies have identified abnormal expression of LncRNAs in disease states, but their role in causing the disease was unclear. Lukiw et al. provided the first report of LncRNA data in aging and neurodegenerative diseases using short post-mortem interval Alzheimer's disease dementia [34]. Subsequently, many studies on LncRNA affecting AD in different ways were reported. For example, LncRNA SOX21-AS1 upregulated oxidative stress in injured neuronal cells of AD models and silencing SOX21-AS1 relieves it. The upregulation of LncRNA SOX21-AS1 resulted in oxidative stress injury of the neurons in the AD model, while the silencing of SOX21-AS1 relieved it [35]. In addition, the upregulated expression of LncRNA EBF3-AS and BDNF-AS promoted neuron apoptosis and oxidative stress in AD [36, 37]. In this study, the LncRNA expression analysis showed 285 differentially expressed LncRNAs. Among them, 242 LncRNAs had co-expression interactions with 82 mRNAs. The co-expression analysis of these LncRNAs and AD-related genes network analysis revealed six important LncRNAs: RP24-454N4.2, LncRNA:chr5:93080503-93084589, A930030B08Rik, Firre, BC055308, and C130013H08Rik. Interestingly, six significant differentially expression mRNAs (SH3BP1, RhoA, RGS4, ST8SIA1, GJA1, and PTMS) were correlated with these lncRNA. These proteins play important roles in signaling pathways for neuronal plasticity and memory formation. The small GTPase mediated signal transduction, known to be involved in cytoskeletal dynamics and intracellular vesicle trafficking, are crucial in synapse/spine formation and remodeling [28, 38]. *SH3BP1*, a Rac1, Cdc42, and TC10-specific GTPase activating protein (GAP) protein, inactivates RAC1 and/or CDC42 allowing the reorganization of the underlying actin cytoskeleton required for synapse/spine remodeling [39]. Regulator of G protein signaling (RGS) family members are regulatory molecules that act as GAPs for G alpha subunits of heterotrimeric G proteins. RGS4 is required for dopaminergic control of striatal LTD [40]. It is strategically positioned to regulate not only postsynaptic but also presynaptic signaling in response to synaptic and nonsynaptic GPCR activation, having broad yet highly selective influences on multiple aspects of PFC cellular physiology [41]. ST8SIA1 is a Ganglioside GD3 synthase and GD3 is required for proper dendritic and spine maturation of newborn neurons in adult brain through the regulation of mitochondrial dynamics [42]. Our results also showed that DSS treatment could rescue cognitive function in APP/PS1 mice and increase the synaptic protein level. It provided clues to the therapeutic effect of DSS for further study of AD target molecules.

To investigate the function of differentially expressed LncRNAs, we constructed the functional analysis with the co-expressed genes of LncRNA and mRNA by using GO and KEGG pathway analyses. The GO terms enrichment results indicated that ion transport, protein ubiquitination, intracellular signal transduction, neuron migration, memory, learning, long-term synaptic potentiation, and regulation of glutamatergic synaptic transmission played important roles in AD development for the DSS treatment group. Several previous articles have reported similar conclusions. Hu et al. [43] reported that DSS could improve the reduction of long-term potentiation and prevent spatial cognitive impairment in SAMP8 mice by reducing the deposition of β-amyloid. Kou et al. [7] used naturally aged mice to study the therapeutic effect of DSS on AD. The results showed that DSS improved memory dysfunction and regulated monoamine neurotransmitter metabolism. DSS has been shown to improve memory damage in the hippocampus associated with acetylcholine and dopamine neurotransmitters [44]. The KEGG pathway enrichment results indicated that the Wnt and Ras signaling pathways, glycerophospholipid metabolism, phosphatidylinositol signaling system, long-term depression, and glutamatergic, dopaminergic, and cholinergic synapses might explain the role that LncRNAs play in DSS treatment to potentially mediate AD pathogenesis.

In conclusion, we identified dysregulated expression profiles of LncRNAs and mRNAs in the hippocampus of APP/PS1 mice that may be potential biomarkers or drug targets relevant to the therapeutic effect of DSS on AD.

## MATERIALS AND METHODS

### Ethics statement

The Hunan University of Chinese Medicine (Changsha, China) institutional review committee provided approval for this research. This study was conducted in accordance with the ethical standards and the Declaration of Helsinki, as well as national and international guidelines.

### Animals

APP/PS1 double transgenic mice (B6C3-Tg; APPswe, PSEN1dE9) 85Dbo/J were purchased from Nanjing Junke Biotechnology Corporation, Ltd. (Nan Jing City, Jiang Su Province, China). The experimental animal production license was No. SCXK (SU) 2017-0003. The animals were housed in a specific pathogen-free animal room of the Hunan University of Chinese Medicine. The breeding method was male hybrid APP/PS1 mice × female wild type C57BL/6J mice (+/-♂×-/-♀), and the feeding proportion was 1:2 (♂:♀). The genotypes of the mice were determined by PCR analysis of genomic DNA from tail biopsies with gene-specific primers. All animals were housed in separate cages (n=5 per cage) at a constant temperature of 25 °C and fed with a standard rodent diet and water with a 12h light/dark cycle. Degenerative neuropathy was evaluated in 7-month-old animals by the water maze test. Once behavioral changes were observed, APP/PS1 mice (n=5) were randomly selected for identification by immunochemistry. APP/PS1 mice were randomly divided into two groups (APP/PS1 group and DSS group) with 10 animals in each group. The DSS group was administered DSS at a dose of 150 mg/kg twice daily via the intragastric route, while the APP/PS1 mice group was administered isopycnic ddH_2_O. The treatment duration was 8 weeks.

### Drugs

DSS of *AngelicaeSinensis Radix* (120 g), *Paeoniae Radix Alba* (640 g), *Poria Cocos* (Schw.) Wolf.(160 g),*Atractylodesmacrocephala*Koidz.(160 g), *Alisma Orientale* (Sam.) Juz. (320 g), and *Chuanxiong Rhizoma*(320 g) was approved by The First Affiliated Hospital of Hunan University of Chinese Medicine (Changsha, China). The six herbs of DSS were mixed and soaked eight times (v/w) in distilled water for 1.5 h, then boiled for 0.5 h, and simmered for 1 h. Then, the filtrate was collected, and the residue was extracted again following the steps described previously, except for rinsing only six times (v/w) in distilled water. The aqueous extract of DSS was concentrated by a rotating evaporator to a final concentration of 1g/ml (equivalent to the dry weight of raw materials).

### Quality control of DSS

An Agilent 1290 Infinity HPLC coupled to an Agilent 6460 Tripe-Quadrupole mass spectrometer equipped with an electrospray ionization interface (Agilent Technologies, Inc. CA, USA) was used for liquid chromatography-tandem mass spectrometry analysis. Chromatographic separation was performed on an Agilent Poroshell 120 EC-C18 (100 × 2.1 mm, 2.7 μm particles) (Agilent Technologies, Inc. CA, USA). Chromatographic and mass spectrometry conditional protocols were performed in accordance with previously described methods [45]. Ferulic acid, paeoniflorin, ligustilide, atractylenolide I, and alisol B 23-acetate (SF8030, SP8030, SL8120, SA8650, Solarbio, China, and YZ-111846, National Institutes for Food and Drug Control, Beijing, China) were used for preparing quality control standards. Standards of 1 mg/ml were prepared and diluted to obtain a standard calibration solution. A total of 0.2 g DSS freeze-dried powder (accurate to 0.0001g) was accurately weighed, dissolved in 2.0 ml 50% methanol, and centrifuged at 10000 r/min for 5min. The supernatant was absorbed to prepare the DSS test solution. The DSS test solution and standards were filtered with a 0.25 μm-filter membrane and tested on the machine of Agilent liquid mass chromatograph. Instrument control, data acquisition, and quantification were performed using MassHunter Workstation software B. 04. 00 (Agilent Technologies, Inc. CA, USA).

### Genotypes

Tail biopsies (5 ml) were collected, and genomic DNA was extracted using a TIANamp Genomic DNA Kit (DP304, TIANGEN Biotech, China), and Taq PCR Master Mix was prepared using a Taq DNA Mix Kit (KT201, TIANGEN Biotech, China). Genotypes for APP and PS1 repeats were determined by PCR as previously described [14]. In brief, PCR amplifications were performed using the PS1 primers, 5′-AATAGAGAACGGCAGGAGCA-3′ (forward) and 5′-GCCATGAGGGCACTAATCAT-3′ (reverse), under the following protocol: 3 min at 95 °C, followed by 35 cycles of 95 °C for 15 s, 60 °C for 30 s, 72 °C for 30 s, and 72 °C for 5 min. The amplified products were analyzed by the electrophoresis of agarose gel with an ethyl bromide ingot.

### Morris water maze test

The Morris water maze test protocol was conducted as described previously [46]. Briefly, the Morris water maze consists of a circular pool filled with water. The ceiling of the room contained constant visual cues for direction. The collected spatial data included placing the mice facing the wall in the north, east, south, and west positions, and the escape platform was hidden in the middle of the northeast quadrant with water level less than 1 cm. In each test, the mouse was allowed to swim until it found the hidden platform, or until 60 s later, it was guided to the platform, where it remained for 10 s before being sent back to the cages with tissues to dry. The SMART 3.0 System (Panlab, Spain) recorded the escape latency and swim path length four times per day for 5 days. On the final day at the end of the acquisition phase, a test was performed by removing the platform and placing the mouse facing the north side. The time was recorded for the mouse looking for the previously correct quadrant in a 60-s test. Two hours later, the visible platform test was performed with the escape platform in the middle of the southeast quadrant 1 cm above the water level. A camera was added to the top of the escape platform to record the movements of the mice.

### Immunochemistry

The immunohistochemical analysis protocols have been described previously [47]. Briefly, the mice were anesthetized with pentobarbital sodium and subsequently sacrificed by cervical dislocation. Brain tissue was quickly stripped off onto ice and then perfused with 4% paraformaldehyde. The fixed brain was cut into 50-μm sections using a vibratome (Leica Biosystems Inc., Germany). The primary antibody 6E10 was performed for Aβ deposition (SIG-39320, Covance Inc., USA). Immunohistochemical statistics were performed on five animals in each group; three slices were selected from each animal, and three fields were randomly selected from each slice to calculate the number of amyloid plaques under 4× objective. Immunoreactivity of Aβ in the hippocampus of the mouse brain was quantified using a histomorphometry system (Image-Pro Plus, Media Cybernetics, Rockville, MD).

### LncRNA expression analysis

Total RNA was extracted and processed for the RNA sequencing (APP/PS1 group n=3 and DSS group n=3). Total RNA was extracted using Trizol reagent (Invitrogen, CA, USA) following the manufacturer’s procedure. The total RNA quantity and purity were analyzed by a Bioanalyzer 2100 and RNA 6000 Nano LabChip Kit (Agilent, CA, USA) with the RIN number >7.0. Approximately 10 μg of total RNA representing a specific adipose type was used to deplete ribosomal RNA according to the manuscript of the EpicentreRibo-Zero Gold Kit (Illumina, San Diego, USA). Following purification, the poly(A)- or poly(A)+ RNA fractions were fragmented into small pieces using divalent cations under elevated temperature. Then, the cleaved RNA fragments were reverse-transcribed to create the final cDNA library in accordance with the protocol for the mRNA-Seq sample preparation kit (Illumina, San Diego, USA). The average insert size for the paired-end libraries was 300 bp (±50 bp). LC-Bio (Hangzhou, China) performed the paired-end sequencing on an Illumina Hiseq 4000 following the vendor’s recommended protocol. LC-Bio (Hangzhou, China) performed all LncRNA expression analyses.

### Differential expression analysis of mRNAs and LncRNAs

StringTie [48] was used to measure the expression levels of mRNAs and LncRNAs by calculating fragments per kilobase of exon model per million reads mapped. The differentially expressed mRNAs and LncRNAs were selected with a fold change ≥ 2 and statistical significance (*P* < 0.05) by the R package Ballgown [49].

### Co-expression network of LncRNAs and mRNAs

The regulation network of LncRNA regulatory genes was analyzed according to the Pearson correlation coefficient of genes and LncRNAs. Then, the LncRNA-mRNA regulatory network was constructed using Cytoscape software V3.6.1 (San Diego, CA, USA).

### GO and pathway analysis

GO and KEGG pathway analyses were performed to analyze the functions of the differentially expressed genes and LncRNAs, as previously described [50, 51]. In this study, GO term enrichment with *P*< 0.05 and false discovery rate (FDR) < 0.05 were employed. GO terms meeting this condition were defined as significantly enriched GO terms in the gene set. Pathway analysis was used to determine the significant pathway of the differential genes, according to KEGG. The calculated *P* value was determined through FDR correction, using FDR < 0.05 as the threshold. Pathways meeting this condition were defined as significantly enriched pathways in the given gene set.

### qPCR

Total RNA was extracted using TRIzol Reagent (Invitrogen, CA, USA) and then reverse-transcribed into cDNA using a TaKaRaPrimeScript RT reagent Kit with gDNA Eraser (RR047A, Takara, Japan) according to the manufacturer’s procedure. Real-time PCR was performed with SYBR Premix Ex Taq II (RR820L, Takara, Japan) and a CFX96 real-time PCR System (Bio-Rad) according to the manufacturer’s instructions. The specific primers are listed in Table 2. The data represents the means of three experiments.

**Table 2:** Primers of related genes in real time PCR.

**Name**	**Forward Primer**	**Reverse Primer**
Tubb2b	GGCGAGGATGAGGCTTGAGTTC	GGCAACAGTGAAGAGCACCAGA
Arf6	GCTCTGGCGGCATTACTACACC	GGTCCTGCTTGTTGGCGAAGAT
S1pr1	GCCTCCTTGCCATCGCCATT	GAGCAGAGTGAAGACGGTGGTG
Arc	TGTATGTGGACGCTGAGGAGGA	ACTGGCTACTGACTCGCTGGTA
Mgat3	CGCCTTCGTGGTCTGTGAATCT	CGCAGGTAGTCATCCGCAATCC
Ddit4	TCGTCCTCCTCTTCCTCTTCGT	GCCACTGTTGCTGCTGTCCA
A930030B08Rik	ACGGAAGGAAGGTGGTGAGGA	TGAGCAGAAGAGCAGAGTGAGC
BC055308	GGTAAGCACACCACCACTGAGA	TTCACGGCAACTGGTAGCAAGT
RP24-454N4.2	TGGTCCTGCCTGCACTGAGAAT	ACTGGGACAACTGCCCTGATGA
Gm44242	TTCTGACCGCTGTAGGCAACC	CGCTGGTAGTGGTAGTGGAAGA
C130013H08Rik	TTGAAACCAGACGGCGAAACCA	AGCCAGGTGAAGCAGCACTATG
AI504432	TGCCAGCAGACAGACAGAATCA	CTGCCACTGCACTCTCATCCAT
GAPDH	CGGTGCTGAGTATGTCGTGGAG	GGTGGCAGTGATGGCATGGA

### Western blotting

100mg hippocampal tissue was ground and crushed using liquid nitrogen. Total protein was extracted according to the RIPA kit instructions (Beyotime Biotechnology Co., Ltd., cat. P0013B, Shanghai, China). The protein concentration was determined using Pierce™ BCA Protein Assay Kit (Thermo Scientific Technology (China) Co., Ltd., cat. 23227, Shanghai, China). The 20μg total protein was separated by SDS-PAGE and transferred to 0.45 μm PVDF membranes (Millipore Sigma Inc., IPVH00010, Billerica, USA). The membranes were blocked with 5% skim milk and incubated overnight with primary antibodies (1:1000) at 4 °C. The membranes were then incubated with secondary antibodies (1:10000) for 1h at 37 °C. Then, ECL chemiluminescence was used in accordance with the instructions of the ECL Plus™ Western Blotting Substrate kit (Thermo Scientific Technology (China) Co. Ltd., cat. 32132, Shanghai, China), and the signals were collected using an imaging system (ChemiDoc™ XRS+, Bio-Rad, California, USA). The primary antibodies Anti-PSD95 (cat. ab18258) and Anti-Synapsin I (cat. ab64581) were purchased from Abcam (Shanghai, China). The primary antibody β-actin (cat. bs-0061R) were purchased from Bioss Biotechnology Co., Ltd. (Beijing, China). The goat anti-rabbit IgG antibody (cat. AP132P) was purchased from Sigma-Aldrich, Inc. (Billerica, USA).

### Statistical analysis

Statistical analysis was performed using Statistical Product and Service Solutions (SPSS, Version21.0). The two groups were compared using the t-test, whereas repeated measurements were made using a one-way analysis of variance. The threshold was set to FC ≥ 2, and *P*< 0.05 was used for screening differentially expressed mRNAs and LncRNAs.
